# Surface structures with unconventional patterns and shapes generated by femtosecond structured light fields

**DOI:** 10.1038/s41598-018-31768-w

**Published:** 2018-09-11

**Authors:** Jijil JJ Nivas, Elaheh Allahyari, Filippo Cardano, Andrea Rubano, Rosalba Fittipaldi, Antonio Vecchione, Domenico Paparo, Lorenzo Marrucci, Riccardo Bruzzese, Salvatore Amoruso

**Affiliations:** 1 Dipartimento di Fisica “Ettore Pancini”, Università di Napoli Federico II, Complesso Universitario di Monte S. Angelo, Via Cintia, I-80126 Napoli, Italy; 2 CNR-SPIN UOS Napoli, Complesso Universitario di Monte S. Angelo, Via Cintia, I-80126 Napoli, Italy; 3 CNR-SPIN, UOS Salerno, Via Giovanni Paolo II 132, I-84084 Fisciano, Italy; 4 National Research Council, Institute of Applied Science & Intelligent Systems (ISASI) ‘E. Caianiello’, Via Campi Flegrei 34, 80078 Pozzuoli, NA Italy

## Abstract

We present an investigation on ultrashort laser surface structuring with structured light fields generated by various q-plates. In particular, q-plates with topological charges *q* = 1, 3/2, 2, 5/2 are used to generate femtosecond (fs) vector vortex beams, and form complex periodic surface structures through multi-pulse ablation of a solid crystalline silicon target. We show how optical retardation tuning of the q-plate offers a feasible way to vary the fluence transverse distribution of the beam, thus allowing the production of structures with peculiar shapes, which depend on the value of *q*. The features of the generated surface structures are compared with the vector vortex beam characteristics at the focal plane, by rationalizing their relationship with the local state of the laser light. Our experimental findings demonstrate how irradiation with fs complex light beams can offer a valuable route to design unconventional surface structures.

## Introduction

Structured or complex light beams with tailored intensity, polarization or phase have emerged as an interesting route in a variety of applications (e.g., microscopy, quantum optics and information, photonics, optical trapping, data communication, etc.)^1–6^. The development of efficient beam converters allows generating powerful pulses of complex light beams, which is of particular interest in direct laser processing and surface structuring. For example, optical vortex beams with nanosecond pulse duration have been exploited to fabricate chiral micro-needles on various materials (e.g. metals, silicon and azo-polymer)^7,8^, while a variety of structures have been achieved with fs pulses (e.g., silicon nano-cones^9^, polymer micro-tubes^10^, graphene micro- and nano-disks^11^, nano-cavities in glasses^12^, etc.). An amazing property of fs laser pulses is the ability to trigger the formation of self-organized quasi-periodic surface patterns, generally indicated as laser-induced periodic surface structures (LIPSS)^13–15^. Under fs laser irradiation, LIPSS with a period Λ considerably smaller than the laser wavelength, λ, or with a near-wavelength period are formed^13^, which are generally termed as *high spatial frequency* LIPSS (HSFL, typ. Λ ≪ λ/2) and *low spatial frequency* LIPSS (LFSL, Λ ~ λ), respectively. In the present investigation, with LIPSS we namely refer to LFSL. For metals and semiconductors, the typical LIPSS are ripples perpendicular to the laser polarization forming a surface grating with a sub-wavelength pitch. The ripples formation is generally interpreted as resulting from an interference process between the incident laser light and a surface scattered wave associated to surface roughness^16–19^, but also other mechanisms, like self-organization of surface instabilities, hydrodynamics, etc., can be involved^20–22^. More recently, for semiconductors (e.g. InP and Si), other LIPSS characterized by a supra-wavelength period and a preferential orientation along the laser polarization, indicated as grooves, have attracted attention^23,24^. Grooves are typically formed at locations characterized by higher fluence, and for large number of laser pulses; their genesis seems to be strictly related to a further spatial modulation of the intensity appearing at higher excitation level, and to the presence of nanoparticles back-deposited on the target surface during the ablation process^25–27^. However, upon multiple-pulse irradiation the periodic intensity modulations progressively lead to local ablation and/or material reorganization, eventually leading to LIPSS formation on the material surface.

**Figure 1:**
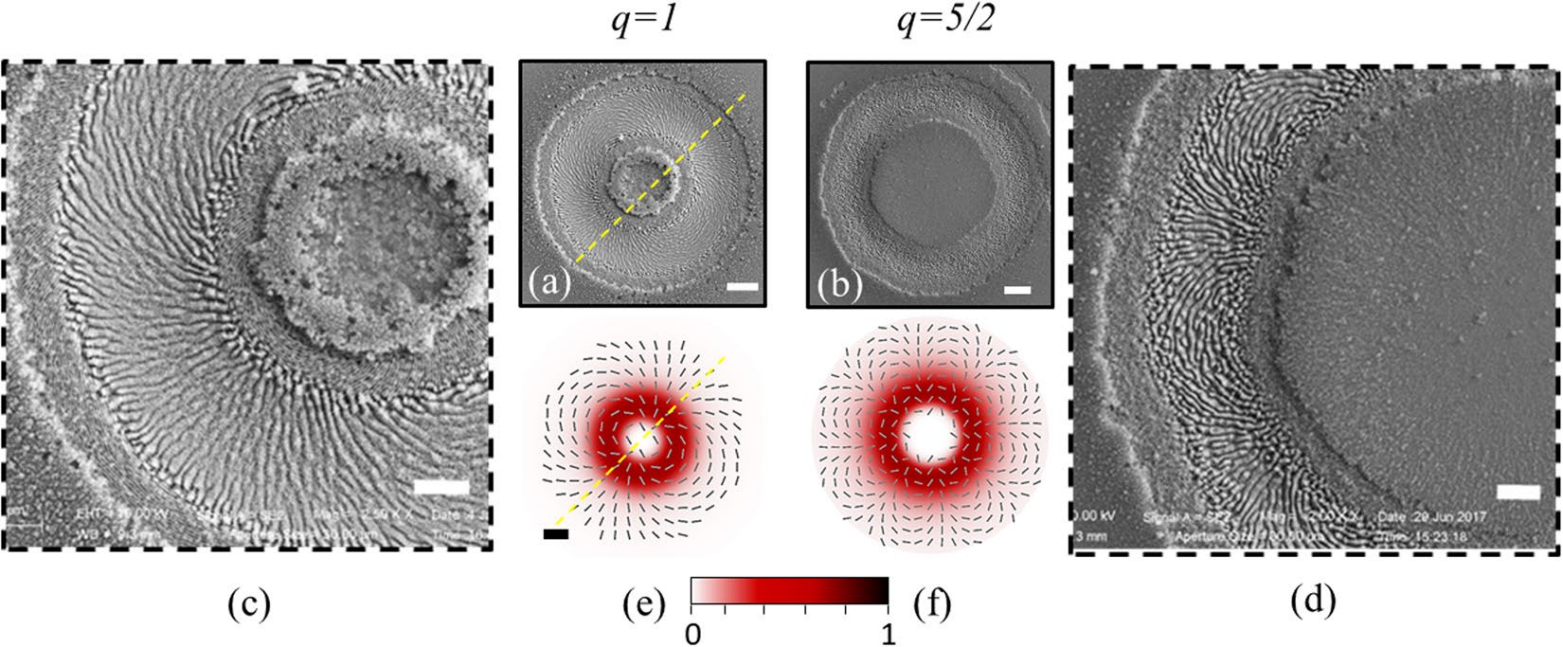
Panels (a) and (b): SEM images showing the surface morphologies developed on the silicon target after an irradiation sequence of *N* = 200 pulses in tuned condition of q-plates (*δ* = π), for (**a**) *q* = 1, and (**b**) *q* = 5/2, respectively. The pulse energy is *E*_0_ = 50 µJ for *q* = 1 and *E*_0_ = 100 µJ for *q* = 5/2. Panels (c) and (d) display zoomed views of the SEM image of panels (a) and (b), respectively. The scale bars in SEM images are 20 µm for (a) and (b) and 10 µm for (c) and (d). Panels (e) and (f) report simulation of far-field beam profile with local direction of the beam polarization. In the maps, each beam intensity profile is normalized to its own maximum value and shown in the color bar, while the spatial scale bar for the maps, shown in panel (e), is 20 μm. The yellow dotted line in panel (a) marks a direction along which grooves alignment closely resembles a quasi-radial pattern, while in panel (e) it shows the corresponding line in the SoP of the beam. On either sides of this dotted lines, the surface structures and SoP of the beam are arranged as a family of spiral-like patterns.

The general characteristics that the LIPSS orientation is linked to the state of polarization (SoP) of the incident beam has been exploited to obtain new morphological patterns by generating ripples and grooves with fs vector vortex (VV) beams characterized by spatially variant polarization and intensity distributions^26,28–31^. Moreover, the analysis of the surface structures has also been shown to offer a useful approach to the characterization of intense complex light fields^32–35^. Most of the previous studies deal with symmetric beams with ring intensity distribution and simple polarization state (e.g. linear, radial, azimuthal, spiral)^26,28,30,32–34^, while more complex light fields like VV with higher topological charge, or composite spatial intensity profiles are still rarely investigated^29,31^. In the present work, we focus on laser-induced surface structures formed on a crystalline silicon target through multi-pulse irradiation with fs VV laser fields produced by q-plates with various topological charges. The q-plate is a thin optical device based on liquid crystal technology^4,36^ that allows generating light beams with phase or polarization singularities^37^. A q-plate essentially works as an inhomogeneous birefringent linear retarder characterized by a distribution pattern of the local optic axes in the transverse *x-y* plane. The local optic axis is oriented with respect to the *x* axis at an angle *α* given by the equation:

1 $$\alpha (r,\varphi )=q\varphi +{\alpha }_{0}$$

where $\varphi =arctan(\frac{y}{x})$ is the azimuthal angle, $r=\sqrt{{x}^{2}+{y}^{2}\,}\,$is the radial coordinate and ${\alpha }_{0}$ is the initial orientation angle at *ϕ* = 0. Besides the topological charge *q*, the action of the q-plate is determined by the value of the birefringent optical retardation *δ*. The q-plate cell has a homogeneous thickness, hence *δ* is uniform point by point along the plate and its value is controlled through an external electric voltage applied to the plate^38^. The q-plate transforms an input light field described by the Jones vector ${{\boldsymbol{\psi }}}_{in}(x,y)$ into an emerging light field ${{\boldsymbol{\psi }}}_{out}(x,y)\,\,$given by:

2 $${{\boldsymbol{\psi }}}_{out}(x,y)=[cos(\frac{\delta }{2})I-i\,sin(\frac{\delta }{2}){M}_{q}(\alpha )]{{\boldsymbol{\psi }}}_{in}(x,y)$$

where *I* is the identity matrix, and ${M}_{q}(\alpha )$ is the Jones matrix of a half-wave plate whose axis forms an angle *α* with respect to the reference *x*-axis. Equation (2) shows that the optical retardation, *δ*, acts as a tuning parameter between two limiting states. For the un-tuned condition, *δ* = 2π, the q-plate behaves as a transparent medium leaving unchanged the input beam. At the optimal tuning, *δ* = π, it converts the input beam into a more complex optical beam capable of carrying an orbital angular momentum (OAM)^37^ (see section Methods). Irradiating the q-plate with a linearly polarized input beam allows generating a superposition of left and right circularly polarized helical beams carrying opposite values of OAM, *ℓ* = ±2*q* (i.e. a null total OAM), and characterized by topologically non-trivial polarization patterns (see e.g. Fig. 1, panels (e) and (f)). The output beam presents a spatial intensity distribution characterized by a central region of zero intensity (due to undefined phase on the beam axis), a principal intense annulus and several secondary rings at increasing radial distance from the axis^4,26^. The most intense part of this beam is spatially filtered with an iris, thus converting the input beam into a beam with an annular spatial profile and a complex spatial distribution of the polarization. We show that such kind of complex light fields can lead to the generation of surface structures with rather complex distribution of ripples and grooves. In addition, by tuning the value of the optical birefringent retardation of the q-plates, *δ*, other light fields with a composite intensity and polarization distribution can be produced. Our findings indicate that these complex light beams offer a further degree of freedom allowing one to imprint surface structures with peculiar shapes related to the value of the q-plate topological charge.

## Results and Discussion

The surface structuring experiments are carried out by focusing the generated beams on the surface of an intrinsic (100) silicon target, in air, with a number of pulses, *N*, selected by an electromechanical shutter. The experimental setup is illustrated in detail in the section Methods. Briefly, as input beam to the q-plate we use a Gaussian laser beam with a pulse duration of ≈35 fs, at a central wavelength of 800 nm, that is linearly polarized along the reference (horizontal) *x*-axis, i.e. ${{\boldsymbol{\psi }}}_{in}(x,y)={{\boldsymbol{G}}}_{H}(x,y)$. Here we exploit four different q-plates with *q* = 1, 3/2, 2 and 5/2. Hence, in tuned conditions (*δ* = π), the silicon target is irradiated by a VV beam ${{\boldsymbol{V}}}_{q}(x,y)={M}_{q}(\alpha ){{\boldsymbol{G}}}_{H}(x,y)$ that is a superposition of two helical beams with opposite OAM *ℓ* = ±2*q*, except for a multiplicative phase term ${e}^{-i\frac{\pi }{2}}=-\,i$. Examples of the spatial distribution of intensity and polarization of the ${V}_{q}(x,y)$ beams are shown in Fig. 1. By varying the optical retardation *δ* we produce a superposition state of the ${{\boldsymbol{G}}}_{H}$ and ${{\boldsymbol{V}}}_{q}$ light fields given by:

3 $${{\boldsymbol{\psi }}}_{out}(x,y)=cos(\frac{\delta }{2}){{\boldsymbol{G}}}_{H}(x,y)-i\,sin(\frac{\delta }{2}){{\boldsymbol{V}}}_{q}(x,y)$$

For each value of the topological charge *q*, Eq. (3) represents an ensemble of complex VV light fields characterized by an inhomogeneous distribution of both polarization and fluence, of which ${{\boldsymbol{G}}}_{H}(x,y)\,\,$and ${{\boldsymbol{V}}}_{q}(x,y)$ are the two degenerate cases. Some novel and interesting aspects of direct fs laser surface structuring carried out by such a kind of structured light beams generated with a q-plate with *q* = 1/2 on a silicon target were addressed in a previous communication^26^. Here, we further assess and extend the possibilities offered by direct laser surface structuring with complex light exploring various surface structures generated by using q-plates with a higher topological charge. The produced surface structures are compared with the polarization and intensity distributions of the VV beams at the focal plane of the lens. We illustrate first the surface structures produced by using the VV beams generated at optimal tuning conditions (*δ* = π). Then, we address the role of the variable optical retardation, finally presenting and discussing surface structures with peculiar shapes, that are generated with appropriate selection of the q-plate and of the optical retardation *δ*.

### Surface structuring at optimal tuning (*δ* = π)

Here, we discuss the surface structures produced by electrically tuning the q-plates to the standard optical retardation of *δ* = π, so as to achieve a conversion to an annular VV beam (see Eq. 3 and Fig. 1). Figure 1 reports examples of SEM images of the target surface and maps of the spatial profiles of both fluence and polarization for two VV beams, namely those produced with *q* = 1 and *q* = 5/2. In particular, panels (a),(b) of Fig. 1 illustrate the morphology of the crystalline silicon surface after irradiation with *N* = 200 pulses at a pulse energy *E*_0_ = 50 µJ for *q* = 1 and *E*_0_ = 100 µJ for *q* = 5/2. The lateral panels (c) and (d) are zoomed views of the SEM images of panels (a) and (b), respectively, evidencing the LIPSS. Finally, panels (e) and (f) report maps of the beam intensity and polarization. In the maps, each beam intensity profile is normalized to its own maximum value.

**Figure 2:**
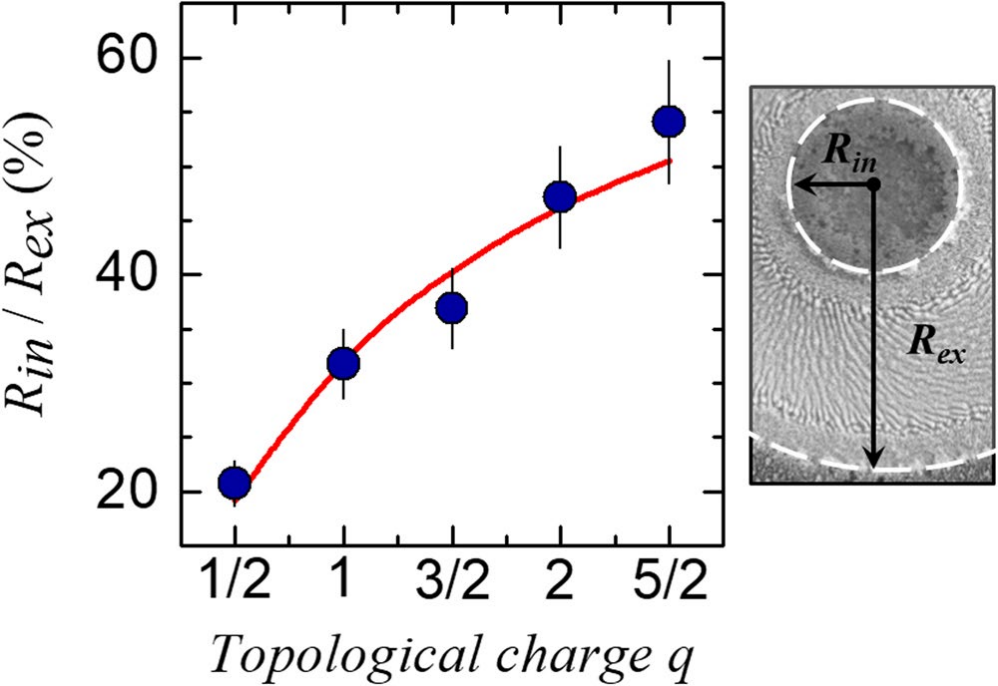
Variation of the ratio between the internal and external radii, *R*_in_/*R*_ex_, for the craters produced by *N* = 200 laser pulses with the VV beams generated by various q-plates at an optical retardation *δ* = π, as a function of topological charge *q*. The symbols are experimental data points. The solid line represents the expected trend obtained by fixing an appropriate ablation threshold value coherent with the experimental data. The error bars are standard error estimated by repeated measurements of the area of the internal and external circles of the annular crater, and propagating the uncertainties to the radii ratio. The right inset visualizes *R*_*in*_ and *R*_*ex*_ on a SEM image. The values of the laser pulse energy are *E*_0_ = 30 µJ for q = 1/2, *E*_0_ = 50 µJ for q = 1, *E*_*0*_ = 100 µJ for q = 3/2, 2 and 5/2. As ablation occurs above a given threshold fluence value at a fixed number of pulses, the ratio *R*_*in*_/*R*_*ex*_ is independent of the specific value of the pulse energy, namely depending on the shape of the spatial profile and, hence, on the value of *q*.

The VV beams clearly produce craters with an annular shape resembling their intensity distribution. The actual value of external and internal radii of the annular craters depend on the specific value of the pulse energy used in our experimental conditions. However, the SEM images clearly evidence that the relative fraction of the central region unaffected by the ablation process increases with the topological charge *q*, as required by the corresponding increase of the low fluence central region of the beam. In an attempt to underline the consistency between the experimental results and the beam intensity profiles, we estimated the ratio of the internal and external radii measured at a fixed value of the pulse number *N* = 200, for all the q-plates. The experimental data are shown in Fig. 2 as solid dots. Moreover, we also insert the corresponding data point for *q* = 1/2 as obtained in our previous investigation in similar experimental conditions^29^. The ratio *R*_*in*_/*R*_*ex*_ clearly increases with the topological charge *q*, passing from ≈20% at *q* = 1/2 to ≈55% at *q* = 5/2. Since the removal of material in the ablation process is a threshold phenomenon, the expected ratio between the radii can be derived from the maps of the fluence spatial profiles by fixing an appropriate fraction of the peak fluence coherent with the experimental case. The corresponding values are reported as a solid, red line in Fig. 2. The comparison shows that the expected behavior reproduces fairly well the observed experimental trend, thus supporting the good consistency between the features expected according to the VV beam spatial profiles and the main characteristics of the annular craters formed on the silicon target.

In all cases, the central non-ablated region is covered with an assembly of a large number of nanoparticles, as typically observed during fs laser surface structuring in air with annular VV beams^26,31^. These nanoparticles form during the material decomposition ensuing fs laser irradiation and target ablation, and are back-deposited on the sample surface due to the effective confinement of the ambient pressure^27^.

We turn now to the analysis of the LIPSS formed within the annular craters. The SEM images of Fig. 1 display the rather complex surface patterns generated by VV beams. In all cases, three annular regions characterized by different surface morphologies are recognized. The central ring, corresponding to the region of higher laser fluence and presenting supra-wavelength grooves, and two adjacent annuli on both sides, covered by sub-wavelength ripples. Taking as an example the simpler case of *q* = 1 (Fig. 1(a)), one can observe that the overall spot is characterized by an annular ablation crater with external and internal radii *R*_*ex*_ (*q* = 1) ≈ 66 µm and *R*_*in*_ (*q* = 1) ≈ 22 µm. The central part of this crater is covered by an annular region of grooves with a width of ≈29 µm surrounded by two rippled areas, at the external and internal boundaries, whose widths are ≈9.5 µm and ≈7.5 µm, respectively. The different width of the rippled regions is due to the slightly dissimilar variation of the beam intensity spatial profile at the two sides of its peak position. The average period of the ripples and grooves measured in the SEM images of Fig. 1(a) are (535 ± 36) nm and (1.7 ± 0.2) µm, respectively, in agreement with previous observations^26^. The variation of the LIPSS period on laser pulse energy and number of pulses follows a behaviour similar to that already discussed earlier both for VV beams^26^ and more standard Gaussian beams^19,21,23,39,40^. Hence, our investigation does not identify any direct influence of the q-value on the LIPSS period, rather the main effect of the different q values is on the various LIPSS spatial patterns that can be generated, which are namely related to the spatially-varying polarization and fluence of the VV laser beams.

**Figure 3:**
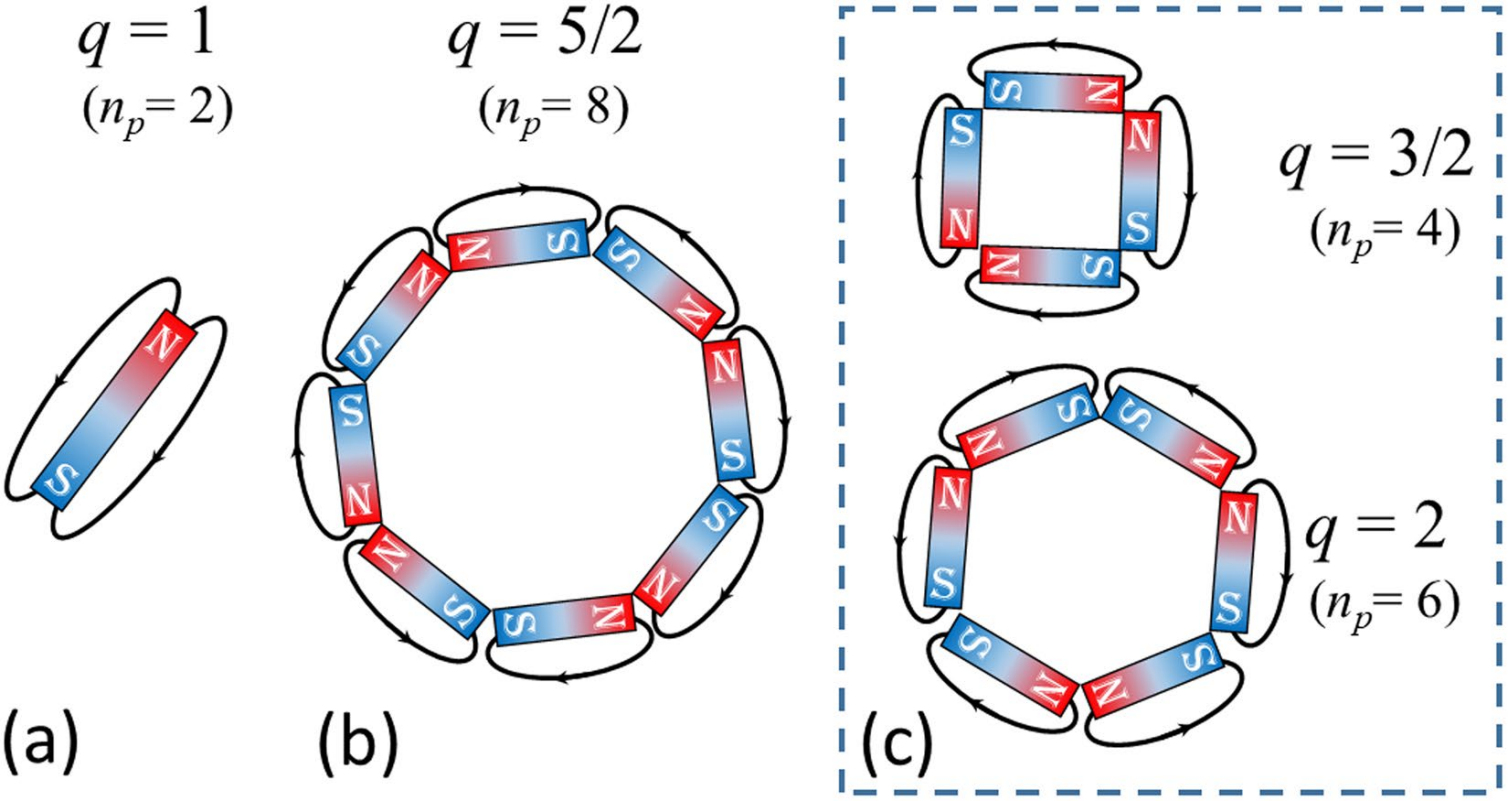
Panels (a) and (b) report schematic illustrations of the spatial distribution in-plane magnetic field lines generated by an arrangement of bar magnets graphically resembling the spatial distribution of the grooves formed by the VV beams generated by q-plates in tuned conditions (*δ* = π) and shown in Fig. 1(a,b). The right inset (panel c) shows the same kind of schematic for the other q-plates used, namely *q* = 3/2 and *q* = 2. Interestingly, the number of independent polar vertices locations *n*_*p*_ corresponds to *n*_*p*_ = 2 (|ℓ| − 1) = (4*q* − 2).

Interestingly, the polarization-locking nature of LIPSS allows a direct visualization of the local polarization direction providing a spatial map of the state of polarization (SoP) of the beam engraved on the target surface. However, the more complex nature of the SoP does not make it possible to identify exact radial or azimuthal patterns as for the simpler case of *q* = 1/2^26,31^. Nonetheless, in the map of the beam SoP (Fig. 1(a), lower panel) one can easily discern two symmetrical areas, located along the direction indicated by the yellow dotted line, characterized by a nearly radial polarization direction, and recognize the corresponding regions of the crater where grooves alignment closely resembles a radial pattern, while ripples approximately follow an azimuthal distribution. On either sides of this dotted line, LIPSS are arranged as a family of spiral-like patterns. Graphically, in panel (a) of Fig. 1 the grooves resembles the spatial distribution of the in-plane magnetic field lines generated by a bar magnet kept along the direction of the yellow dotted line. As the value of *q* increases, the SoP of the VV beam and, consequently, the spatial distribution of the surface features become progressively more and more complex. However, following the same kind of illustration used before, one can qualitatively describe the spatial orientation of the grooves patterns produced by the VV beam with *q* = 5/2 as in-plane magnetic field lines generated by eight bar magnets arranged as in Fig. 3(b) with alternating *N* and *S* poles. Interestingly, the number of independent polar vertices locations *n*_*p*_ corresponds to *n*_*p*_ = 2(|*ℓ*| − 1). This is confirmed also for the other *q* values, for which the representative sketches are shown in Fig. 3(c). This pictorial representation suggests that the grooves formed on the silicon target surface provide an easy and direct visualization of the beam SoP also for more complex beams, thanks to their inherent property of orienting along the field polarization. Even if the larger dimensions of the supra-wavelength grooves may somewhat limit the spatial resolution in comparison to sub-wavelength ripples, they clearly offer a more straightforward picture of the SoP in weak focusing conditions, i.e. for beam spot sizes in the range of tens of microns. Moreover, in many practical conditions, the generation of uniform grooves is a comparatively easier phenomenon, that takes place in the higher intensity region of the beam or for larger number of pulses^23,24,26,27,41^, while ripples are confined in narrower regions around the low intensity beam edge where fluence values are closer to the ablation threshold.

### Surface structure produced at variable optical retardation *δ* by beams generated by a q-plate with *q* = 1

The q-plate beam converter easily allows generating a variety of complex light beams in the form of a superposition of the VV beam obtained at tuned conditions (*δ* = π) illustrated earlier, and the input Gaussian beam. As indicated by Eq. (3), the fractional contribution of the two components depends on the optical retardation *δ*, which is easily controlled by an external voltage^4,38^. Hereafter, we illustrate the effect of the optical retardation tuning on the surface structures imprinted on the silicon target for several *δ* values in the case of *q* = 1. Then, selected examples of the surface structures produced by VV beams at higher values of the topological charge *q* will be discussed in the next section, thus addressing the versatility of the q-plate approach to laser texturing with fs VV beams.

**Figure 4:**
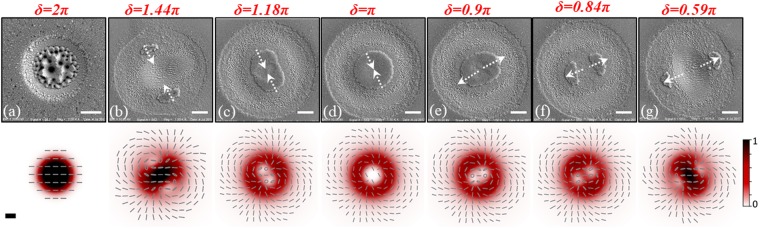
Panels (a–g) show SEM images of the silicon surface after irradiation with VV beams generated by q-plate with *q* = 1 at different values of the optical retardation *δ* ranging from *δ* = 2π (Gaussian beam) to *δ* = 0.59π (number of laser pulses *N* = 200, pulse energy *E*_0_ ≈ 40 µJ). For the sake of completeness, panel (d) shows the case of a tuned q-plate (*δ* = π) corresponding to a VV beam with |ℓ| = 2, discussed in the previous section. In each SEM image, the dotted arrows mark the direction along which the regions of minimum intensity of the VV beam move while varying the optical retardation *δ*. The scale bar in each SEM image corresponds to 20 µm. For each case, the lower panel displays the corresponding spatial distribution of the VV beam intensity and SoP. The scale bar, shown in lower left map, is 20 μm.

The upper panels of Fig. 4 report SEM images of the silicon target surface after irradiation with VV beams generated by the q-plate with *q* = 1 at different values of the optical retardation *δ*, ranging from *δ* = 2π to *δ* = 0.59π. For the sake of completeness, also the tuned case (*δ* = π), corresponding to the VV beam with |*ℓ*| = 2 discussed earlier, is reported as panel (d). The experimental conditions used are *N* = 200 laser pulses at an energy *E*_0_ ≈ 40 µJ. The corresponding SoP and intensity maps of the VV beams are shown in the lower panels of Fig. 4.

Let us consider first the case of the Gaussian beam ${G}_{H}$ at *δ* = 2π in panel (a), comparing it with the features of the modifications induced by the VV beam ${V}_{q}\,\,$at *δ* = π of panel (d) already discussed earlier. The Gaussian beam ${G}_{H}\,\,$produces a smaller crater with an external radius of ≈31 μm, while the corresponding VV beam ${V}_{q}\,\,$generates a shallower annular crater with a larger external radius and an overall width of (37.6 ± 0.8) µm. Moreover, while in the case of the VV beam, ripples decorate either sides of the grooved central annular region (22 ± 1) µm wide, the morphology of the crater induced by the Gaussian beam is rather different. This diverse texture is due to the fact that the present analysis is carried out at a fixed energy *E*_0_ ≈ 40 µJ, which in the case of ${G}_{H}\,\,$is spread over a smaller spot than for ${V}_{q}$, thus leading to a higher peak fluence, as also indicated by the intensity maps of Fig. 4 (lower panels). The peak fluence of the Gaussian beam at the beam centre (*x* = 0, *y* = 0) is $\frac{2{E}_{0}}{\pi {w}_{0}^{2}}\approx 1.4$ J/cm^2^, which is about 2.7 times larger than that of peak fluence value $2{{\rm{e}}}^{-1}\frac{E}{\pi {{w}_{0}}^{2}}\approx 0.51$ J/cm^2^ of the ${V}_{q}$ beam with |*ℓ*| = 1^42^, occurring at a radial position *r*_*p*_ ≈ 30 μm. At the higher peak fluence achieved by the Gaussian beam, the crater is characterized by two principal regions presenting different surface textures. Coarse wrinkles decorated with several columnar structures and deep cavities cover the central area of the crater. This kind of surface structures typically forms in high laser fluence regions, or after a large number of pulses^13,24,29^. The central area is surrounded by grooves covering a ≈ 9.5 µm wide annular ring. The grooves are aligned along the laser polarization direction and are characterized by an average spatial period of (1.6 ± 0.2) µm. Besides the grooves, no clear signature of ripples is recognized in the SEM image of Fig. 4(a). This is likely due to a very limited region of ripple formation, and to a dense re-deposition of nanoparticles generated in the fs ablation around the circular crater. In fact, craters produced by Gaussian beams at reduced laser peak fluence, in similar experimental conditions, have shown a progressive reduction of the central area characterized by the coarse structures, followed by the annular grooved region and, eventually, surrounded by an external rippled area^24^.

We turn now to illustrate the effects of the optical retardation tuning. As an example, panels (c) and (e) of Fig. 4 report SEM images for *δ* = 1.18π, and *δ* = 0.9π, i.e. for *δ* slightly changed from the optimal tuning *δ*_*opt*_ = π for the VV beam generation. One can observe that the central circular region of minimum fluence produced at *δ*_*opt*_ progressively transforms into an elliptical area, in correspondence of even little detuning from the condition of optimal tuning. In particular, the orientations of the major axes of the two ellipses depend on the sign of (*δ* − *δ*_*opt*_) and are orthogonal to each other (see panels (c) and (e) of Fig. 4). Panels (b) and (f) show the further variations occurring when the optical retardation reaches values of *δ* = 1.44π and *δ* = 0.84π, respectively. In these cases, the ellipses separate in two regions located at larger distance from the crater centre. These two regions tend to move further apart along two orthogonal directions as |*δ* − *δ*_*opt*_| increases, eventually reaching the external border of the annular crater. It should also be noted that the total area of the two regions of minimum fluence, below the ablation threshold, is much less than both the elliptical area before splitting, and the area of the central disk at optimal tuning. Moreover, splitting and migration of the regions of minimum fluence is accompanied by a variation of the ripple and groove orientations, as well as of the general surface texture, as a consequence of the modifications in the spatial distribution of laser fluence and SoP due to the progressive change in the contributions of the two fields, i.e., ${G}_{H}\,\,$and ${V}_{q}$ terms of Eq. (3). The observed evolution of the surface texture agrees fairly well with the variation of the fluence spatial distribution reported in the lower panels, showing that as *δ* progressively moves from *δ*_*opt*_, the central region of minimum intensity splits into two separate regions and the surface structure changes following the spatial distributions of the complex beam SoP and fluence. The observed direction along which the region of minimum fluence moves depends on the input polarization direction and on the value of *α*_0_, which in the present case is estimate to be *α*_0_ ≈ −π/6. Splitting of the minimum fluence region in two parts is a consequence of the decay of the central high-order optical vortex, associated with the two circular components, into elementary ones. This qualitatively justify the direct relationship between the number of split regions and the charge of the q-plate^43^. Finally, these experimental findings further confirm that direct fs laser surface texturing with complex light beams generated by a q-plate, assisted by the possibility of optical retardation tuning, offers an effective route to the fabrication of rather composite surface features and LIPSS. This aspect is further illustrated in the following section by reporting some examples of peculiar surface structures generated with VV beams produced by q-plates with higher values of the topological charge *q*.

### Peculiar surface structure produced by complex light beams

**Figure 5:**
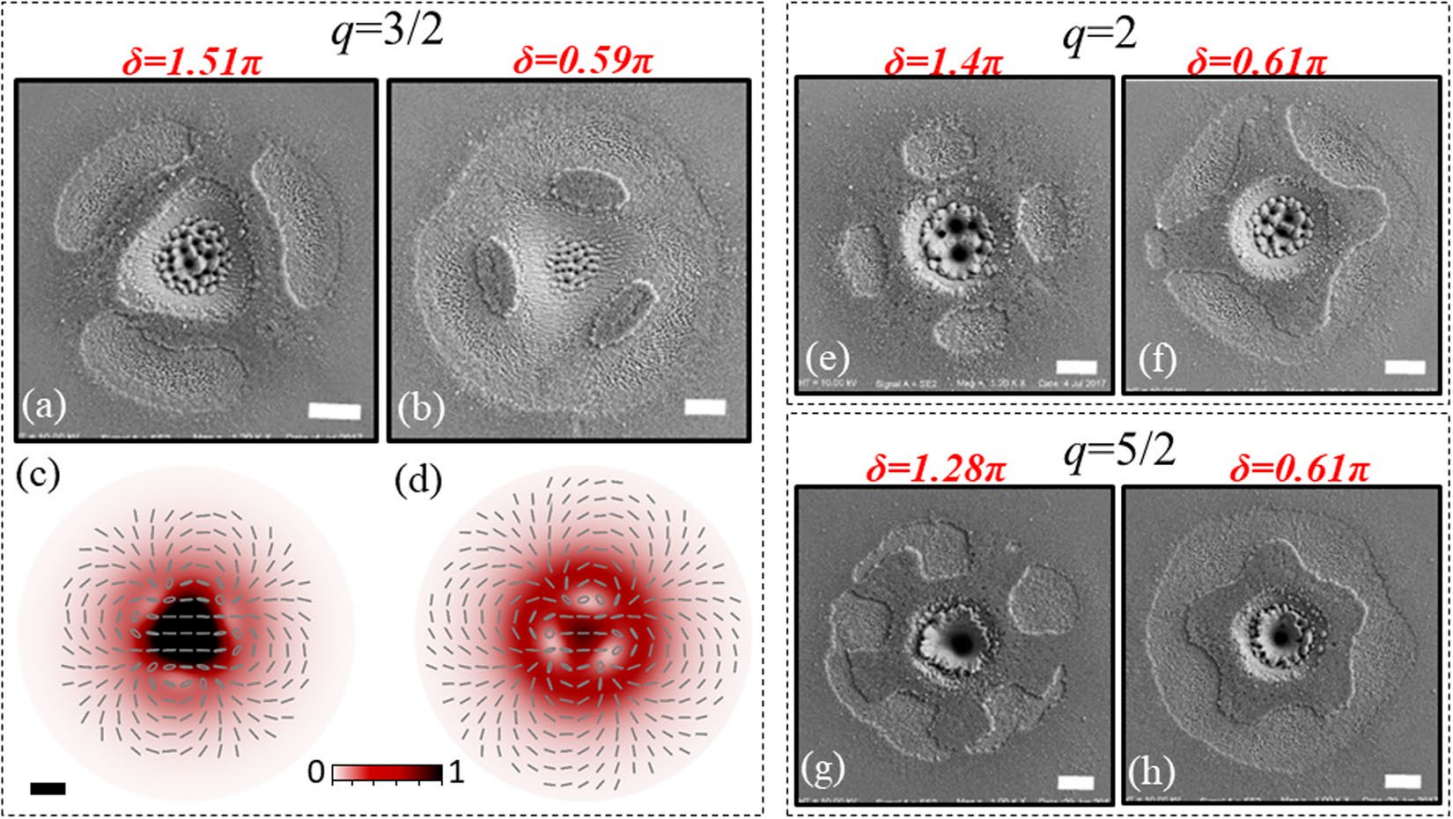
SEM images illustrating the structures formed on the silicon surface after irradiation with fs VV beams generated by three different q-plates for two different values of the optical retardation *δ*. The scale bar in the SEM images corresponds to 20 µm. Panels (a) and (b) show the case of *q* = 3/2 for *δ* = 1.51π and *δ* = 0.59π at a pulse energy *E*_*0*_ = 65 µJ. For this case, the lower panels (c) and (d) display the corresponding simulated far field intensity and SoP patterns of the corresponding VV beams. The scale bar, shown in panel (c), is 20 μm. Panels (e) and (f) illustrate the case of *q* = 2 for *δ* = 1.4π and *δ* = 0.61π at a pulse energy *E*_*0*_ = 55 µJ. Panels (g) and (h) report the case *q* = 5/2 for *δ* = 1.28π and *δ* = 0.61π at a pulse energy *E*_*0*_ = 100 µJ. In all cases, the number of pulses is *N* = 200.

Figure 5 reports some SEM images exemplifying the variety of patterns that can be imprinted on the silicon sample surface by using VV beams generated by q-plates with a larger topological charge *q*, namely *q* = 3/2, 2 and 5/2, through an appropriate selection of the optical retardation *δ*. In particular, in Fig. 5 two conditions corresponding to *δ* below and above the optimal retardation value (*δ*_*opt*_ = π) are shown for each q-plate. For *q* = 3/2, the lower panels (c) and (d) display the corresponding spatial distribution of the VV beam intensity and SoP.

We illustrate first the case *q* = 3/2 reported in panels (a) and (b) of Fig. 5. For *δ* = 1.51π, Fig. 5(a) shows a pattern composed by three individual, arc-shaped, ablated lobes, located on a circular track at ≈120° from each other, surrounding a triangular central region. This form is fairly consistent with the spatial distribution of the laser fluence of the VV beam, shown in Fig. 5(c). The VV beam intensity profile is characterized by a central intense region, due to the ${G}_{H}$ component, surrounded by a less intense external part coming from the annular ${V}_{q}$ contribution. In particular, it presents a spatial modulation on the external ringed region enclosing the central triangular high-intensity part, with three maxima and minima located at about 120° from each other, explaining the three ablated lobes. A closer look to the various parts composing the pattern reveals the presence of LIPSS. In particular, each one of the single, external lobes shows ripples and grooves with a preferential orientation, in fairly good agreement with the SoP of the VV beam shown in Fig. 5(c). Instead, the peripheral area of the central region is decorated by nearly horizontal grooves resembling the linear polarization of the predominant ${G}_{H}$ contribution, meanwhile columnar structures typically formed at higher fluence cover the center of the triangular crater. A complementary form is imprinted on the sample surface for *δ* = 0.59π, as shown in Fig. 5(b). In this case, the pattern is characterized by three elliptical non-ablated islands located at 120° from each other, and surrounded by an ablated area presenting a variety of micro/nano structures. Also in this case, the imprinted form agrees well with the fluence spatial distribution of the VV laser beam of Fig. 5(d), showing three regions of very low fluence within a quasi-circular spot with a central intensity peak. Again, the central region is characterized by a triangular intense part, with vertices lying in the zones separating the elliptical islands, and presenting linear grooves and columnar structures, resulting from the dominant ${G}_{H}$ component with horizontal polarization. Moreover, the rest of the crater is covered by LIPSS with a complex spatial arrangement reflecting the intricate SoP of the VV beam. This example illustrates the complex morphology that can be obtained with VV beams generated by optical retardation tuning of a q-plate with *q* = 3/2; interestingly, the triangular shape of the central region, as well as the three ablated lobes at *δ* = 1.51π, and the three non-ablated islands at *δ* = 0.59π address the OAM of the constituent helical beams forming the ${V}_{q}$ component, i.e. |*ℓ*| = 3.

We turn now to *q* = 2 with the two examples of surface patterns reported in panels (e) and (f) of Fig. 5, corresponding to *δ* = 1.4π and *δ* = 0.61π, respectively. At *δ* = 1.4π (Fig. 5(e)), a central crater is surrounded by four elliptical ablated regions located at the vertices of a square. An optical retardation of *δ* = 0.61π (Fig. 5(f)) leads to the generation of four main non-ablated regions that are linked by bridges left in the less intense zones of the beam encircling the central, high-fluence area. Hence, the non-ablated part resembles a paddle wheel inscribed in the irradiated area and presenting a central hole. The two upper-left arms of the paddle are joined in some points with the external sample surface, possibly because of slight local non-uniformities of the laser beam fluence profile at the edges of the ablated area. Finally, for *q* = 5/2 the two interesting shapes shown in panels (g) and (h) of Fig. 5 are produced at *δ* = 1.28π and *δ* = 0.61π, respectively. The morphology of the non-ablated parts in Fig. 5(g) resembles a paddle wheel with 5 arms (or a flower with five petals) with a pentagonal shaped hole in the center, where the ${G}_{H}\,$component is dominant. Instead, Fig. 5(h) shows the formation of a starfish-like shaped feature with a deep central crater. Both *q* = 2 and *q* = 5/2 confirm a remarkable relation of the characteristics of the generated patterns with the OAM of the helical beams forming the ${V}_{q}$ component, i.e. |*ℓ*| = 4 and 5, respectively. Moreover, in both cases, the orientation of the LIPSS imprinted in the ablated regions is consistent with the SoP of the VV beam.

**Figure 6:**
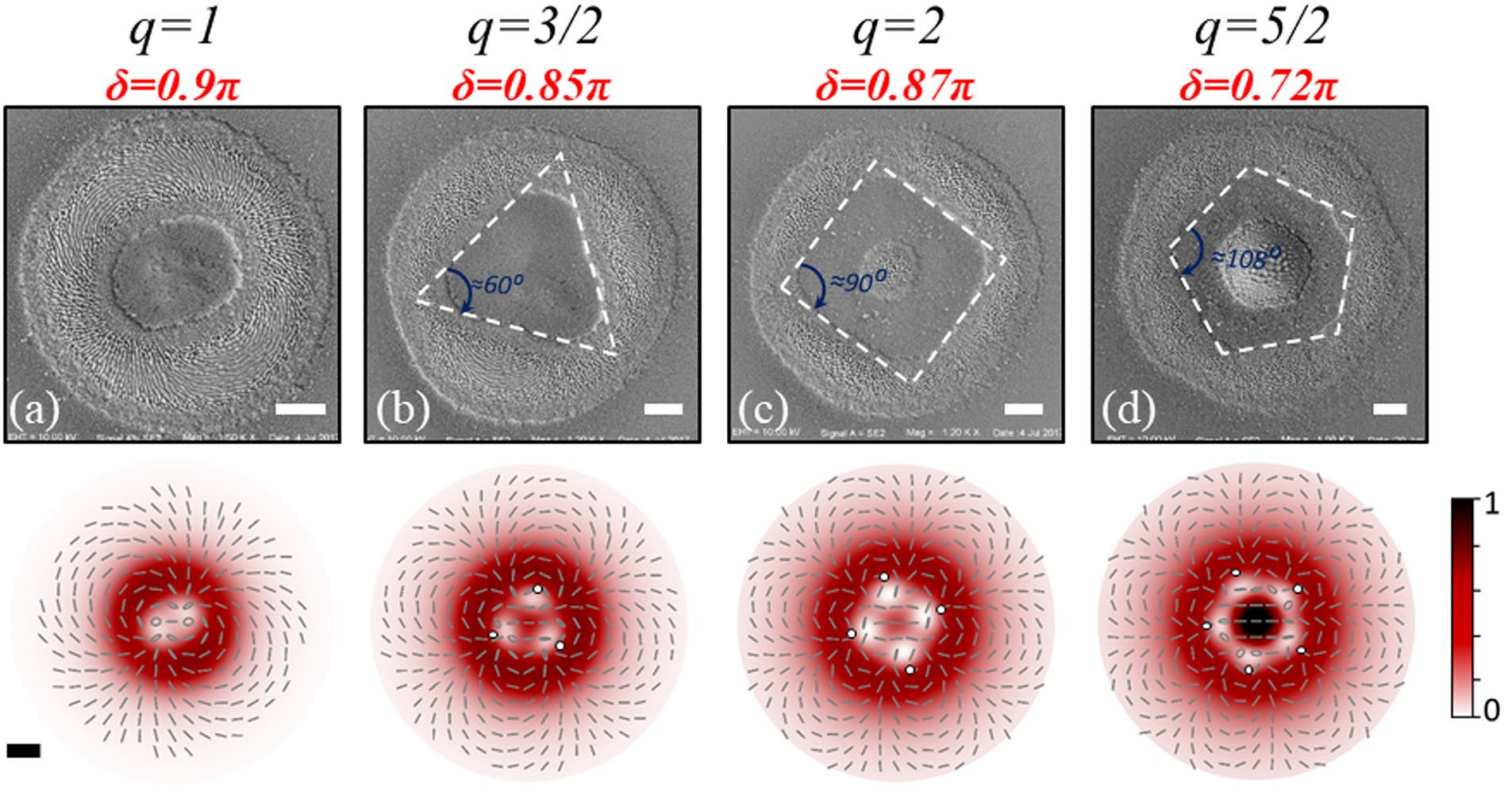
SEM images of the silicon surface after irradiation with N = 200 fs VV pulses generated by four different q-plates; the topological charge, q, and optical retardation, *δ*, are indicated on the top of each image. The scale bars in the images correspond to 20 µm. The structures are produced at a laser pulse energy of: (**a**) 40 µJ, (**b**) 65 µJ, (**c)** 55 µJ, and (**d**) 100 µJ, respectively. The lower panels display the spatial maps of the fluence and SoP of the corresponding VV beam. The scale bar, shown in lower left map, is 20 μm.

Finally, Fig. 6 reports SEM images of some very peculiar structures obtained with VV beams that further emphasize the potentiality and versatility of their use in direct fs laser surface processing. In particular, here we consider the non-ablated area remaining in the central part of the crater, whose shape results from the spatial modulation of the VV beam fluence, and the existence of a definite threshold for material removal in the laser ablation process. Therefore, the structures have been achieved by tailoring the laser fluence spatial distribution of the VV beams, through the optical retardation of the q-plates, and their corresponding intensity maps are displayed in the lower panels of Fig. 6. As already discussed above, for *q* = 1 the spatial modulation of the laser fluence by optical retardation tuning leads to two regions of minimum fluence (see e.g. Fig. 4). When the distance between these regions is not enough to lead to separated non-ablated islands, as, e.g., for *δ* = 0.9π (Fig. 6(a)), an elliptical non-ablated area is obtained. At *q* = 3/2, three regions of minimum fluence form in the VV beam spatial profile, as for example illustrated in Fig. 5(b). Also in this case, an appropriate optical retardation tuning can lead to a spatial fluence distribution limiting the ablation in the central area and allowing to imprint a blunt triangle in the center of the ablation crater, as for example shown in Fig. 6(b) at *δ* = 0.85π. To facilitate the identification of the resulting shape, three white dotted segments along the straight edges of the central non-ablated part are drawn in Fig. 6(b). The internal angles at the three vertices formed by these lines are (61 ± 1) degrees, hence showing that an equilateral blunt triangle is eventually achieved. The corresponding three vertices are indicated as small circles in the map of the fluence spatial distribution reported in the lower panel of Fig. 6(b).

Increasing the value of q-plate topological charge *q*, blunt square and pentagonal patterns are achieved for *q* = 2 at *δ* = 0.87π and for *q* = 5/2 at *δ* = 0.72π, respectively. The mean of the internal angles between straight edges in Fig. 6(c) and (d) are (90 ± 2) and (107 ± 2) degrees, respectively. However, in this cases one can also observe the formation of a circular ablated area within the central regions. The central hole is due to the gradual increase of the intensity of the ${G}_{H}$ component at the spot center, as evidenced by the spatial intensity maps of the VV beams (see lower panels of Fig. 6(c) and (d)). These examples suggest that appropriate optical tuning of the q-plates allows generating VV beams capable of scribing peculiar geometrical shapes on the sample surface that are characterized by a fairly good degree of regularity. The number of sides of the imprinted figure is directly related to the OAM, |*ℓ*|, carried by the left and right circularly polarized helical beams forming the ${V}_{q}$ component, going from a segment with rounded extremes at |*ℓ*| = 2 to a blunt pentagon at |*ℓ*| = 5. Surface structures with such morphologies may attract particular interest because of their potential to impart novel functionality and responses to the material surface. In fact, the basic units can be replicated over larger areas by laser step scan processing. This might allow the fabrication of arrays of structures with high precision and reproducibility, and in short time, through direct laser surface processing with tailored VV beams.

## Conclusions

In summary, we have presented an experimental analysis on fs laser surface structuring with complex VV beams generated by q-plates with topological charges *q* = 1, 3/2, 2, 5/2, through multi-pulse ablation of a solid crystalline silicon target. Our findings show good agreement between the spatial profile of fluence and SoP of the beam in the focal plane, and the features of the generated ablation craters and LIPSS (ripples and grooves) patterns. In particular, we have demonstrated that optical retardation tuning of the q-plate offers a novel strategy to manipulate the fluence distribution of the beam, and fabricate specific surface structures, with peculiar shapes. This is achieved by simply tuning the voltage to the q-plate, thus making our approach very useful and advantageous as a beam shaping technique since it allows generating a variety of fields in an easy way and with the use of a flat and compact optical element. A variety of shapes imprinted on the sample surface has been observed, that comprehends reliefs of non-ablated regions in form of isolated islands, multi-ablated spots with particular geometries, hollowed paddle wheels, starfish and blunt regular polygons (e.g., triangle, square and pentagon). We have, thus, demonstrated that the use of ultrashort complex light beams in direct surface structuring allows achieving a further advance in laser patterning and texturing. The features of the generated surface structures are compared with the vector vortex beam characteristics at the focal plane rationalizing their relationship with the local state of the laser light. In conclusion, irradiation with fs complex light beams can offer a valuable route to design unconventional surface structures, which, moreover, can provide basic units that replicated over larger areas allow fabricating complex surfaces with novel or extended functionality.

## Methods

### Experimental setup

**Figure 7:**
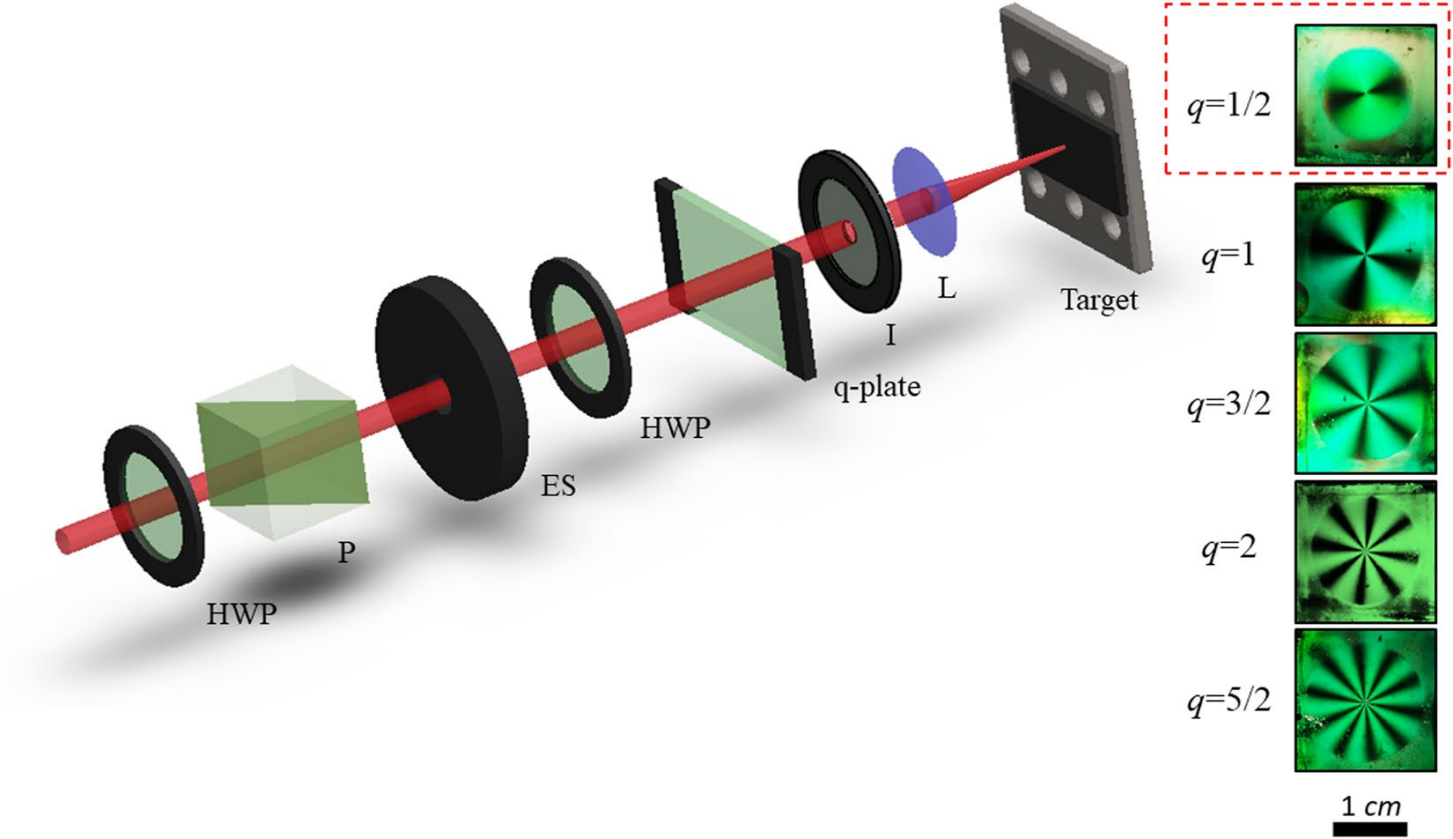
Schematics of the experimental setup used for fs laser surface structuring with VV beams generated by a q-plate. HWP = half-wave plate, P = polarizer, ES = electro-mechanical shutter; I = iris; L = lens. The silicon target is fixed on a computer controlled high precision 3-axis translation stage. The right panel shows images of the q-plates used in our experiments, e.g. *q* = 1, 3/2, 2, and 5/2, kept in between to cross-polarizing sheets. The image in the red dashed box corresponds to a q-plate with *q* = 1/2.

The experimental setup is sketched in Fig. 7. The laser source is a Ti:Sa laser system delivering ≈35 fs pulses at a central wavelength of 800 nm, with a Gaussian beam spatial profile, at a repetition rate of 10 Hz. A combination of half wave-plate and polarizer is used to control the pulse energy. Optical VV beams are generated by a beam converter based on different q-plates with topological charges *q* = 1, 3/2, 2, and 5/2 and an initial angle *α*_0_ ≈ −π/6 (see Eq. (1)). The q-plate tuning is achieved by varying the optical retardation *δ* by means of the driving voltage *V*_*pp*_ (peak to peak) applied to the q-plate, by using a square-wave at 11 kHz delivered by a signal generator^4,38^. At optimal tuning, corresponding to a half-wave retardation (*δ* = π), the q-plate allows generating various complex light fields depending on the polarization of the input beam. By using a linear polarization along the reference (horizontal) *x*-axis, the q-plate generates a superposition state of left and right circularly polarized helical beams, whose spatial distribution in cylindrical coordinates can be expressed as:

4 $${{\boldsymbol{\psi }}}_{VV}^{(\ell )}\propto {f}_{\ell }(r,z)({{\boldsymbol{e}}}_{{\boldsymbol{l}}}\,{e}^{i|\ell |\varphi }+{{\boldsymbol{e}}}_{{\boldsymbol{r}}}\,{e}^{-i|\ell |\varphi })$$

In Eq. (4), $${{\boldsymbol{e}}}_{{\boldsymbol{l}}}$$ and $${{\boldsymbol{e}}}_{{\boldsymbol{r}}}$$ are the unit vectors associated with the left and right circular polarization states, *z* the direction of the propagation axis, $$r$$ and $$\varphi $$ the radial and azimuthal coordinates in the *x-y* plane ($$r=\sqrt{{x}^{2}+{y}^{2}}\,;\,\varphi =$$$$arctan(y/x))$$. The function $${f}_{\ell }(r,z)$$ describes the radial distribution of the field and is identical for the two modes carrying opposite values of the OAM, ±|*ℓ|*. The temporal dependence, inherited from the input laser light pulse, can be treated as a global term not affecting the spatial and polarization structure of the field, and is not shown in Eq. (4). In general, at the exit of a tuned q-plate, i.e. at an optical retardation *δ* = π (see Eq. (2)), the profile of the generated VV beam is described by a Hyper-Geometric Gaussian mode^44,45^. However, by filtering the beam with a circular aperture, we observe that the beam profile is well approximated by a Laguerre-Gauss mode subclass $$L{G}_{0,\ell }$$^5^, which we designate as $${{\boldsymbol{V}}}_{q}(x,y)$$ (see Eq. 3). For conditions of optimal tuning *δ* = π, the VV beam waist at the focal plane is evaluated by the analysis of the variation of the annular crater radii vs laser energy^42^, resulting *w*_0_ ≈ 43 μm. At full wave retardation (*δ* = 2π), instead, the q-plate output results in a Gaussian beam with horizontal polarization $${{\boldsymbol{G}}}_{H}(x,y)$$ (see Eq. (3)). Finally, optical retardation tuning provides a variety of fs VV laser beams (see Eq. (3)) with a rather complex spatial variation of the SoP and fluence distribution.

The surface structuring experiments are carried out by focusing the generated VV beams on polished (100) intrinsic (resistivity > 200 Ωcm) silicon plates (Sil’tronix Silicon Technologies), in air. The number of pulses hitting the target surface, *N*, is selected by an electromechanical shutter (see Fig. 7). The target is an intrinsic (resistivity > 200 Ωcm), single-crystalline Si (100) plate. The laser beam is focused by a lens (focal length 75 mm) onto the Si target sample, mounted on a computer-controlled 3-axis translation stage, at normal incidence. An electromechanical shutter controls the number of laser pulses, *N*, irradiated on the target surface. The morphological modifications of the irradiated target surface are analyzed by means of a field emission scanning electron microscope (FESEM).
